# Editorial Comment: Effect of smoking cessation on sexual function in men aged 30 to 60 years

**DOI:** 10.1590/S1677-5538.IBJU.2019.0541.1

**Published:** 2020-05-20

**Authors:** Carlos Teodósio Da Ros, Fernando Nestor Facio

**Affiliations:** 1 DR&G Urologistas Associados Porto AlegreRS Brasil DR&G Urologistas Associados, Porto Alegre, RS, Brasil.;; 2 Departamento de Andrologia Sociedade Brasileira de Urologia Rio de JaneiroRJ Brasil Departamento de Andrologia, Sociedade Brasileira de Urologia – SBU, Rio de Janeiro, RJ, Brasil;; 3 Departamento de Urologia Faculdade de Medicina de São José Rio Preto São José do Rio PretoSP Brasil Departamento de Urologia, Faculdade de Medicina de São José Rio Preto, São José do Rio Preto, SP, Brasil

## COMMENT

This study involved 181 relatively young individuals (30-60 years) who were former smokers and had no other risk factor for erectile dysfunction (ED) besides smoking. The participants completed the IIEF during the first consultation, while still under the effects of smoking, and again six months later. As expected, the prevalence of ED was significantly lower after smoking cessation ( 1 ).

While much is focused on smoking and its association with cancer and cardiovascular disease, which occur in older individuals, few studies address the effects on young people and adolescents, who can suffer the same adverse effects, including ED. There are approximately one billion smokers in the world and every year eight million people die due to smoking and its adverse effects ( 2 ).

The pathophysiological mechanism of endothelial dysfunction results from an inhibition of the nitric oxide cascade, preventing adequate arterial dilation and the blood flow necessary for penile erection. In addition to endothelial dysfunction, smoking is also a risk factor for arteriosclerosis ( 3 - 5 ). Sahin MO et al., the authors of the study commented here, also mention the correlation between ED and cardiovascular disease and smoking as a risk factor for both ( 1 ).

In smokers with heart disease, the risk of complete ED is seven times higher than that imposed by any of the risk factors alone ( 6 ). In an analysis of more than 31,000 individuals over 50 years of age, the prevalence ED was 33% and was higher among those who were obese, sedentary, smokers and alcoholics ( 7 ). These data were confirmed in several other studies ( 8 , 9 ).

This study offers insight into contemporary indications that smoking is significantly associated with ED and smoking cessation has a beneficial effect on the restoration of erectile function (EF). The literature offers studies showing the association between smoking and hypertension, acute coronary syndrome, angina, atherosclerosis, cerebrovascular diseases and sudden death. Based on this consistency, a fair conclusion may be drawn that male sexual function and smoking have a temporal relation; smoking precedes ED. There is an increased risk of ED with greater exposure to smoking and smoking cessation can lead to the recovery of erectile function but only if lifetime exposure to smoking is limited ( 10 , 11 ).

Based on this study, urologists should counsel smokers with ED to quit smoking, which will result in an improvement in erectile rigidity and tumescence.
